# The role of GPX1 in the pathogenesis of female pelvic organ prolapse

**DOI:** 10.1371/journal.pone.0181896

**Published:** 2017-08-07

**Authors:** Shasha Hong, Li Hong, Bingshu Li, Debin Wu, Cheng Liu, Jie Min, Wenjun Guo, Ming Hu, Jianming Tang, Yang Li

**Affiliations:** Department of Obstetrics and Gynaecology, Renmin Hospital of Wuhan University, Wuhan, Hubei Province, China; Boston University Henry M Goldman School of Dental Medicine, UNITED STATES

## Abstract

Gestation and delivery can increase intra-abdominal pressure, which are well-known risk factors for Pelvic Organ Prolapse (POP). But the pathogenesis mechanism of POP remains unclear. Our previous research showed that the expression of glutathione peroxidase type 1 (GPX1) decreased in pelvic floor ligaments from POP patients, implying that oxidative stress (OS) may be related to POP. The aim of this study was to figure out the role of GPx1 regulation in the pathogenesis of POP. Women (>45 years) who received hysterectomy surgery were enrolled in this research, identified by screening. We applied mechanical strain of 0μ, 5333 μ to GPX1-overexpressing human uterosacral ligament fibroblasts (hUSLFs) isolated from menopausal women without POP respectively for 4 hours, in order to investigate the changes of cell apoptosis, oxidative status and ECM metabolism when cytomechanics model loaded on GPX1-overexpressing hUSLFs. Comparing with the non-transfection and mock-vehicle groups, we found that GPX1 not only protects hUSLFs from cell apoptosis, oxidative damage, but also improves the remodeling of ECM induced by mechanical stimulation. These results suggested that mechanical strain caused abnormalities of ECM metabolism via OS pathway in hUSLFs, which was involved in the pathogenesis of POP, and that GPx1 played a significant role in regulating mechanical strain induced POP.

## Introduction

Pelvic organ prolapse (POP) is a common disease characterized that a loss of normal attachment and support leads to the descent of the pelvic organs into the vaginal canal [1], which affects 50% of all women over the age of 50 years [2]. There are about 226 000 women suffering POP surgery annually in US, and the direct costs of POP therapy are estimated at least 1 billion dollars [3]. POP can cause both financial as well as health burden due to the tremendous costs associated with medical treatment and the high risk of recurrence after surgery. The etiology of POP was poorly defined till recently and there are still gaps in current models.

Mechanical strain has been thought to be the main cause of POP. Gestation and vaginal childbirth, which can enlarge the intra-abdominal pressure and lead to the muscle and fascia trauma, are high risk factors for POP [4, 5]. It is shown that Obesity and increased BMI play an important role in the development of POP with symptomatic POP increasing by 3% with each unit of increasing BMI[4]. Obesity and increased BMI may also contribute to the increased pressure and strain placed on the pelvic floor [6]. Chronic strain is also associated with POP, women who worked as laborers or factory workers got higher rates of severe POP compared with women with more sedentary jobs [7]. However, the exact mechanism of the pelvic mechanical stretches on POP remains unclear.

Current researches mostly focused on uterosacral ligaments (USL) [8, 9], vaginal wall [10–12], and the pubocervical fascia [9, 13] in women with POP. Fibroblasts play an important role in tissue repair and remodeling to maintain tissue homeostasis. In response to strain, extracellular matrix (ECM) senses and transfers external mechanical stimuli into intracellular signaling through cytoskeleton to induce repair and remodeling. Decreases in the biomechanical properties of pelvic supports involving supporting fibroblast and ECM have been thought to cause POP [14]. The main components of ECM in pelvic supporting tissue include collagen I, collagen III, elastin, collagen-degrading enzyme matrix metalloproteinases (MMPs), and tissue inhibitor of metalloproteinases (TIMPs). A decrease in collagen production [15] and elastin content [1], as well as an increase amount of active MMP-2 and MMP-9 [9, 16–17] has been showed in POP patient tissue.

OS(oxidative stress) may be involved in the pathophysiology of POP by contributing to ECM metabolic disorder in a severity-dependent manner in human uterosacral ligament fibroblasts (hUSLFs), possibly through the regulation of MMPs, TIMPs and TGF-β1 indirectly [18]. Oxidative stress-associated factors, including content of isoprostanes [19], DSCR-1 [20], 8-OHdG and 4-HNE [21], were differentially regulated in patients with uterine prolapse, strongly implied the important role of oxidative stress in POP pathology. Furthermore, our previous research also found that excess mechanical stress and H_2_O_2_ inhibited cell proliferation, and decreased mRNA and protein expression levels of ECM components, collagen 1, collagen 3 and elastin in hUSLFs [22], excess mechanical strain increased intracellular ROS levels in hUSLFs [23]. All of above study showed that Mechanical strain caused abnormalities in ECM metabolism via OS pathway, which may participate in the development of POP.

Glutathione peroxidase (GPX) is the general name of an enzyme family with peroxidase activity and protects cells against oxidative damage. It is one of the important indicators to measure the antioxidant ability in human body. Selenium is the essential composition in the activity center of GPX1 enzyme. Se-glutathione peroxidase-1 is the most abundant selenoprotein in cells [24]. There were evidences indicated that GPX is associated with POP. Dimanov [25] described a high prevalence of vaginal or uterine prolapse in buffalo cows having lower blood selenium values compared with other pregnant females. In the study of dromedary camels, data showed the content of serum selenium and glutathione peroxidase in whole blood of the dromedary camels with white muscle disease and uterine prolapsed were lower than that in the dromedary camels only with white muscle disease, and the serum selenium and glutathione peroxidase in the latter were lower than that in the normal dromedary camels, these results suggest that selenium deficiency could promote uterine prolapse in dromedary camels [26]. Our previous research showed that there was a decline in the expression of glutathione peroxidase 1 (GPX1) in USL of women with POP[27]. This suggested GPX1 may play an important role in the pathophysiology of POP. Thus, the present study aimed to study the role of GPX1 in response to mechanical strain, which may provide insight into the pathogenesis of POP.

## Material and methods

### Participants

The present research was performed on human subjects and was approved by the Medical Ethics Committee of Renmin Hospital of Wuhan University (Wuhan, China). All patients gave oral and written informed consent prior to participation in the research. Date of approval 11 September 2012. A total of 10 patients who received hysterectomy surgery for suffering from benign gynecologic disease and in menopausal status were enrolled in this study. One year of amenorrhea in women aged >45 years defined as menopause. None of the recruited women suffered from POP, malignant tumors, connective tissue diseases, pathologically confirmed endometriosis or estrogen‑associated ovarian tumors. Furthermore, the patients were free from any complications that may lead to oxidative stress‑associated diseases, including coronary heart disease, diabetes and hyperlipidemia. Patients who received surgery in the uterosacral ligamental site or had a history of estrogen application within the past three months were also excluded from the present study.

### Primary cell culture

USL tissue specimens were used to develop primary culture of hUSLFs as previously described [28]. Briefly, USL tissues were washed with phosphate‑buffered saline (PBS) and cut to small pieces. The tissues were digested with 1% collagenase‑I (Invitrogen, USA) for 3 h at 37°C in 5% CO_2_, followed by further digestion with 0.25% trypsin (Sigma, USA) for 5 min. 2 ml fetal bovine serum (FBS; Gibco, USA) was used to stop the digestion. Dulbecco's modified Eagle's medium (DMEM; Jenom, Hangzhou, China) containing 15% FBS and penicillin (100 U/ml) and streptomycin (100 μg/ml) (Beyotime, China) was then slowly added to the culture flask. The medium was replaced every two days. Cells released from USL tissues were subcultured when confluence was over 85%. The cells were characterized by their bipolar and spindle-like morphology, and identified as fibroblasts by H&E staining and immunohistochemistry in which results showed strong expression of vimentin and no expression of keratin, as previously described [29]. The hUSLFs were used at passage 3‑8 for the subsequent experiments. Each experiment was repeated in fibroblasts from at least three different donors.

### Cell transfection

The hUSLFs were transfected with an expression vector (GV341, AgeI / NheI enzyme digestion, purchased from Shanghai GeneChem Co., Ltd, China) containing the Lentivirus-GPX1(15640–1). The sequence element of GV341 vector was Ubi-MCS-3FLAG-SV40-puromycin, which was also used as the lentivirus control. Multiplicity of Infection (MOI) was screened to 30 which was confirmed by fluorescent microscope in the preliminary experiment. Briefly, 500 ul LV-GPX1(1*10^9^), 500 ul polybrene and 4ml 15% FBS were added into a 25*25cm2 culture flask at a cell density of 107 cells/ml. After 12 hours transfection, cells were selected by 0.8 *μ*g/ml puromycin supplementation in culture medium and clones were isolated. GPX1 overexpression in cells was controled by Western-Blot. For experiments and results sections, cells were divided into three groups: GPX1 overexpression transfection group(GT), mock-vehicle transfectoion group(MT) and non-transfection group(NT). Transfected cells were always cultured in the presence of 0.8 *μ*g/ml puromycin and 15% FBS culture medium.

### Application of mechanical strain

The fibroblasts in the exponential growth phase at passage 3–8 derived from 10 patients were seeded onto mechanical loading plates which were deformable, transparent and pre-coated with rat tail collagen type I(25 *μ*g/ml in 0.02 N acetic acid) (Sigma, USA). After achieving 75% confluence, fibroblasts were rendered quiescent and incubated in serum-free DMEM for 24h before subjected to mechanical strain. We used a four-point bending device (SXG4201, MIRACLE Technology Co. Ltd, Chengdu, China) to exert mechanical strain on fibroblasts laid on mechanical loading plates. The operating principle and methods of the four-point bending device were described before [29]. In Brief, the device is divided into three parts: Mechanical power systems, host computer and strain loading dish. Parameters were set to a frequency of 0.1 Hz over 4 h, and cells were subjected to strains of 0 *μ*、5333 μ (strain displacement is 0mm、4 mm). The control group samples (0 μ) were incubated at 37°C without any treatment under the same culture conditions.

### JC-1 staining

JC‑1 (Beyotime Institute of Biotechnology) is a sensitive fluorescent probe which can be utilized to measure the mitochondrial membrane potential (ΔΨm). In healthy cells with high mitochondrial ΔΨm, JC‑1 spontaneously forms complexes known as JC‑1 aggregates with intense red fluorescence. By contrast, in apoptotic or unhealthy cells with low ΔΨm, JC‑1 remains in the monomeric form, which shows only green fluorescence. After the fibroblasts had been subjected to the respective strains for 4 h, they were rinsed twice with PBS and incubated with a mixture of 2 μl JC‑1 staining solution and 2 ml serum‑free medium in the dark at 37°C for 30 min according to the manufacturer's instructions. The cells were then washed with JC‑1 staining buffer twice, and 2 ml serum‑free DMEM was added to each specimen. The JC‑1 fluorescence was observed under the inverted fluorescence microscope. Image J software was employed to analyze the red and green fluorescence intensity. The ratio of red to green fluorescence was calculated, which was indicative of the ΔΨm.

### Flow cytometry

The hUSLFs were subjected to strains as mentioned above. Subsequently, the apoptotic rate was determined by Annexin V/PI (Beyotime Institute of Biotechnology) double staining according to the manufacturer's instructions. Briefly, cells from different groups were harvested, washed with ice‑cold PBS twice and re‑suspended in 400 μl binding buffer. 5 μl fluorescein isothiocyanate‑conjugated Annexin V and 10 μl PI were added, followed by incubation for 20 min in the dark at room temperature. The apoptotic rate was analyzed by flow cytometry (BD LSR II; BD Biosciences, Franklin Lakes, NJ, USA) using Flow Jo software 7.6 (BD Biosciences). The cells which stained positive for Annexin V and negative for PI were considered to be early apoptotic, while those which were positive for both were identified as late apoptotic cells. The apoptotic rates were expressed as the percentage of the total cell population.

### Detection of intracellular ROS

The ability of mechanical stress treatment to increase ROS production in hUSLFs was measured by the fluorescent probe 2',7'‑dichlorodihydrofluorescein diacetate (H2DCF‑DA; Beyotime Institute of Biotechnology, Shanghai, China). After subjected to strains, the cells were then washed three times with PBS and incubated for 40 min at 37°C with 1.5 μl H2DCF‑DA in serum‑free medium. Cells were again washed three times with PBS, and fresh serum‑free DMEM was added. Images of cells with ROS‑associated fluorescence were captured using an inverted fluorescence microscope (CKX31; Olympus, Tokyo, Japan), and images were analyzed using Image J version 1.46 software (National Institutes of Health, Bethesda, MD, USA).

### Immunofluorescent staining

8-OHdG and 4-HNE were tested by immunofluorescent assay. Cells plated on the dishes with or without exposure to strain were fixed by 4% paraformaldehyde at room temperature for 20 min, washed with PBS, and permeabilized in 0.3% Triton X-100/PBS for 5 min. After blocking for 1 hour in 5% goat serum, cells were incubated overnight at 4°C with 8-OHdG or 4-HNE(Institute for the Control of Aging, Fukuroi, Japan). Then the dishes were washed in PBS and incubated with secondary antibodies against first antibodies labelled with fluorescent dye. The nuclei were dye with DAPI. The fluorescence-stained cells were observed with fluorescence microscope (IX51, Olympus, Japan).

### Quantitative real-time polymerase chain reaction(qRT-PCR)

Gene expression of COL1A1, COL3A1, Elastin, MMP-2, MMP-9, TIMP-2, TGF-β1 and GAPDH was evaluated by qRT-PCR. The primers used for amplification were purchased from SBS Genetech (SBS Genetech Co., Ltd, Beijing, China). The total RNA of hUSLFs was extracted by using TRIZOL (Invitrogen, USA). Then RNA were reverse transcribed to cDNA. We used SYBR Green labeled probes to detect gene expression in qRT-PCR ABI 7500 System (Applied Biosystems, USA) for in vitro qRT-PCR. The level of mRNA were calculated and normalized to the level of GAPDH mRNA. The primer sequences were used as follows: human GAPDH(Forward 5’-GAAGGTGAAGGTCGGAGTC-3’ and Reverse 5’-GAAGATGGTGATGGGATTTC-3’); human COL1A1(Forward 5’-CAAGACGAAGACATCCCACCAATC-3’ and Reverse 5’-ACA GATCACGTCATCGCACAACA-3’); human COL3A1(Forward 5’-TCGCTCTGCTTCATCCCACTAT-3’ and Reverse 5’-CTTCCAGACATCTCTATCCGCAT-3’); human Elastin(Forward 5’-GGGTTGTGTCACCAGAAGCA-3’ and Reverse 5’-CAACCCCGTAAGTAGGAATGC-3’); human MMP-2(Forward 5’-AGTTTCCATTCCGCTTCCAG-3’ and Reverse 5’-CGGTCGTAGTCCTCAGTGGT-3’); human MMP-9(Forward 5’-GTCCACCCTTGTGCTCTTCC-3’ and Reverse 5’-GACTCTCCACGCATCTCTGC-3’); human TIMP-2(Forward 5'-TCTGGAAAC GACATTTATGG-3' and Reverse 5'-GTTGGAGGCCTGCTTATGGG-3); human TGF-β1 (Forward 5’-TATTGAGCACCTTGGGCACT-3’ and Reverse 5’-ACCTCT CTGGGCTTGTTTCC-3’).

### Western blot

The total proteins were extracted from fibroblasts and then quantified by BCA method (BCA protein assay kit, Beyotime, China). The total proteins from each group were loading in the SDS-PAGE gel and separated by electrophoresis. Then the proteins were transferred onto a PVDF membrane. The membrane was blocked in 5 g/L skim milk for 1 h and washed in TBS. The membrane was incubated in the appropriate monoclonal antibodies at 4°C overnight. After washing in TBS-Tween (TBST), the membrane was incubated with HRP-conjugated anti-IgG secondary antibody (dilution) at 37°C for 1 h. After washing in TBST, the target proteins were visualized by using an ECL detection kit. We used antibodies to the following: GPX1(1:1000, Abcam, UK), COL1A1(1:500, Santa Cruz, USA), COL3A1(1:500, Santa Cruz, USA), Elastin(1:500, Santa Cruz, USA), MMP-2(1:500, Santa Cruz, USA), MMP-9 (1:500, Santa Cruz, USA), TIMP-2(1:500, Santa Cruz, USA), TGF-β1 (1:1000, Abcam, UK), β-actin(1:1000, Abcam, UK) and GAPDH (1:1000, Santa Cruz, USA) were used as an internal reference control.

### Statistical analysis

Data were represented as the mean ± standard deviation (SD). We performed one-way analysis of variance (ANOVA) with Mann-Whitney *U* test for comparisons between two groups. All tests performed using statistical software SPSS 16.0 (SPSS, Inc., Chicago, IL, USA). *P<*0.05 was considered statistically significant. Each experiment was repeated at least three times.

## Results

### 1. Effect of GPX1 overexpression vector transfection

**Fig 1:**
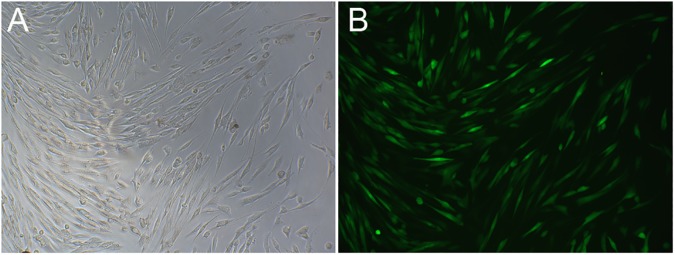
Representative images demonstrating GPX1 transfection efficiency of human uterosacral ligament fibroblasts (hUSLFs). Multiplicity of Infection (MOI) was screened to 30 which was confirmed by fluorescent microscope (B) compared to the bright field (A) (magnification, x 200).

**Fig 2:**
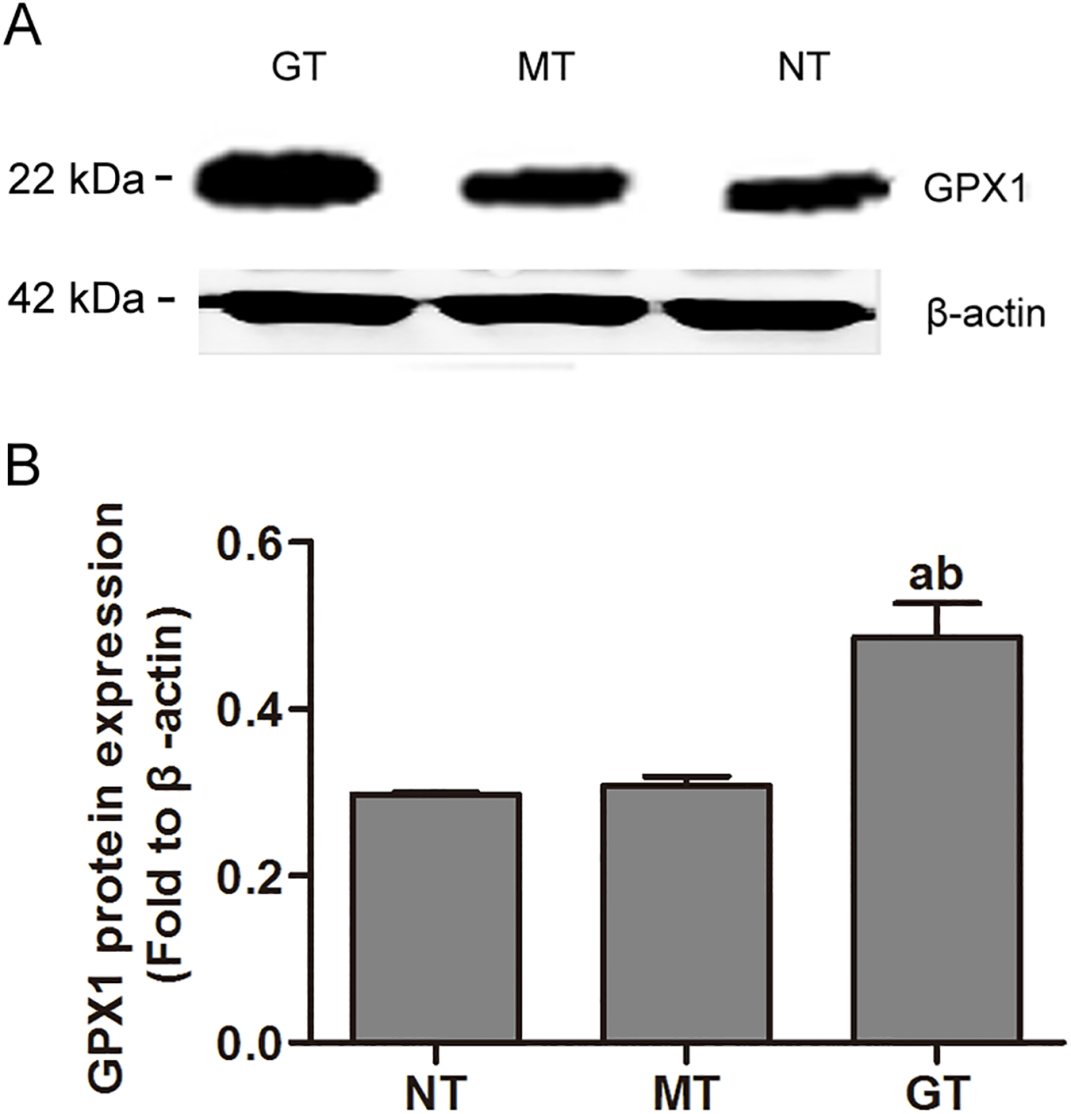
Quantification of GPX1 protein levels. GT, MT and NT cytosolic proteins were subjected to electrophoresis, transferred to a nitrocellulose membrane and immunodetected with anti-human GPX1 antibody. GPX1 protein levels were compared in GPX1-overexpressing group (GT), mock-vehicle group (MT) and non-transfection group (NT) by signal intensity measurement. One-way analysis of variance was performed, followed by an unpaired t-test. n = 3, a, *P*<0.05, vs. 0 μ, NT; b, *P*<0.05, vs. 0 μ, MT; c, *P*<0.05, vs. 5333 μ, NT; d, *P*<0.05, vs. 5333 μ, MT.

In GPX1-overexpressing hUSLFs (MOI = 30) the GFP fluorescence positive rate which represents the cell infection rate was more than 80% (Fig 1). GPX1 protein levels were quantified by Western-blotting. As shown in Fig 2, GPX1 levels did not differ in mock-vehicle group and non-transfection group (respective ratios: 0.307±0.012 and 0.298±0.002). In contrast, in GPX1-overexpressing group, GPX1 protein expression was significantly increased compared to both mock-vehicle and non-transfection groups (ratio 0.485±0.041 vs. 0.307±0.012 and 0.298±0.002 respectively, *P* < 0.01) (Fig 2). All of above results indicated that GPX1 overexpression vector was transfected into hUSLFs successfully as well as effectively in our present study.

### 2. GPX1 protects from mechanical strain induced apoptosis in hUSLFs

**Fig 3:**
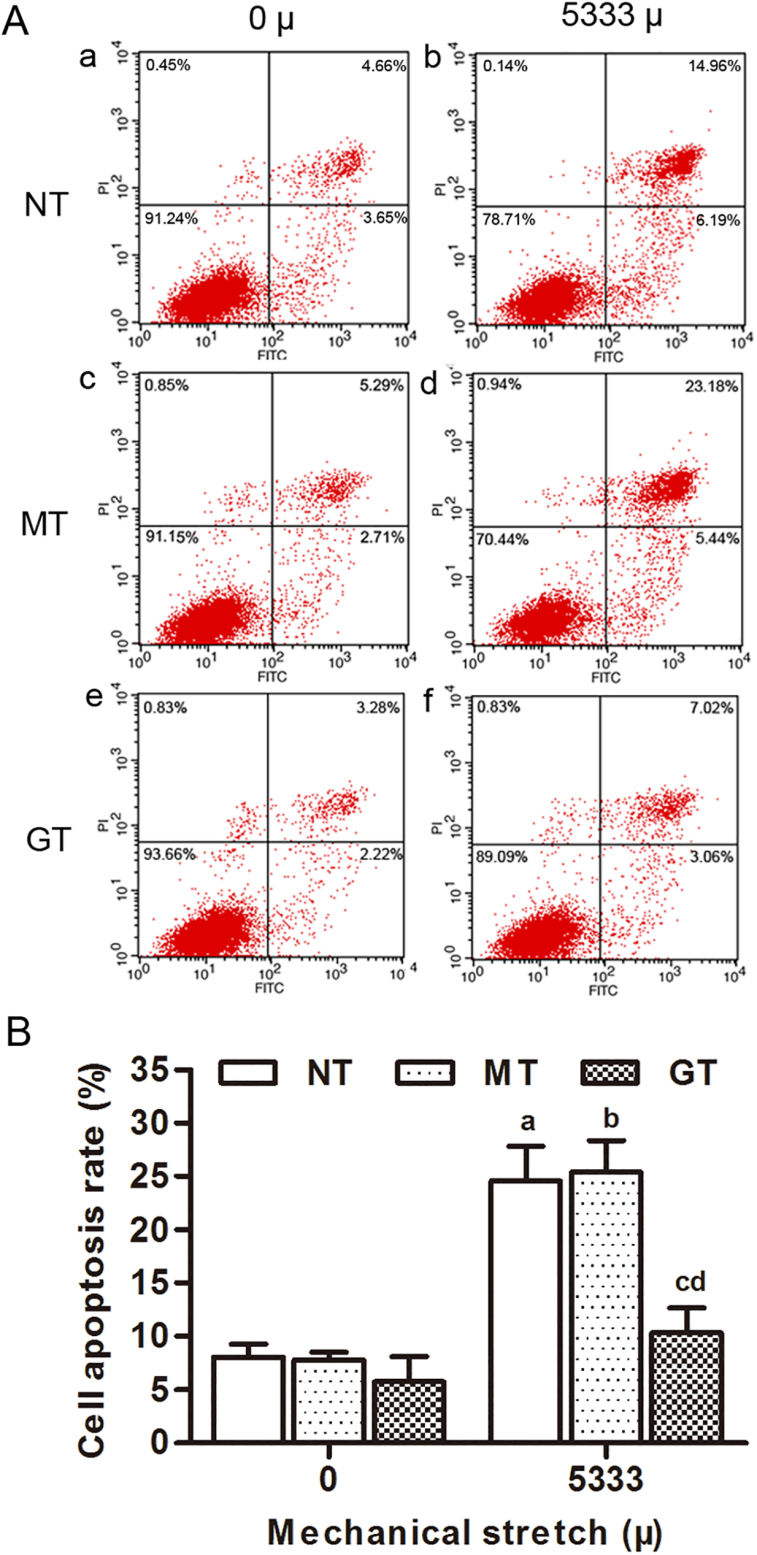
Mechanical stress effected cell apoptosis in hUSLFs. (A) Representative dot plots of cell apoptosis by flow cytometry analysis after Annexin V/PI dual staining. The apoptotic rate was determined as the percentage of Annexin V-positive cells, with early apoptotic cells being PI-negative and late apoptotic cells being PI-positive. (a,b) NT; (c, d) MT; (e, f) GT; (a, c, e) 0 μ strain; (b, d, f) 5333 μ strain. (B) Statistical analysis of the apoptotic rates. Values are expressed as the mean ± standard deviation of three independent experiments. One-way analysis of variance was performed, followed by an unpaired t-test. n = 3, a, *P*<0.05, vs. 0 μ, NT; b, *P*<0.05, vs. 0 μ, MT; c, *P*<0.05, vs. 5333 μ, NT; d, *P*<0.05, vs. 5333 μ, MT.

**Fig 4:**
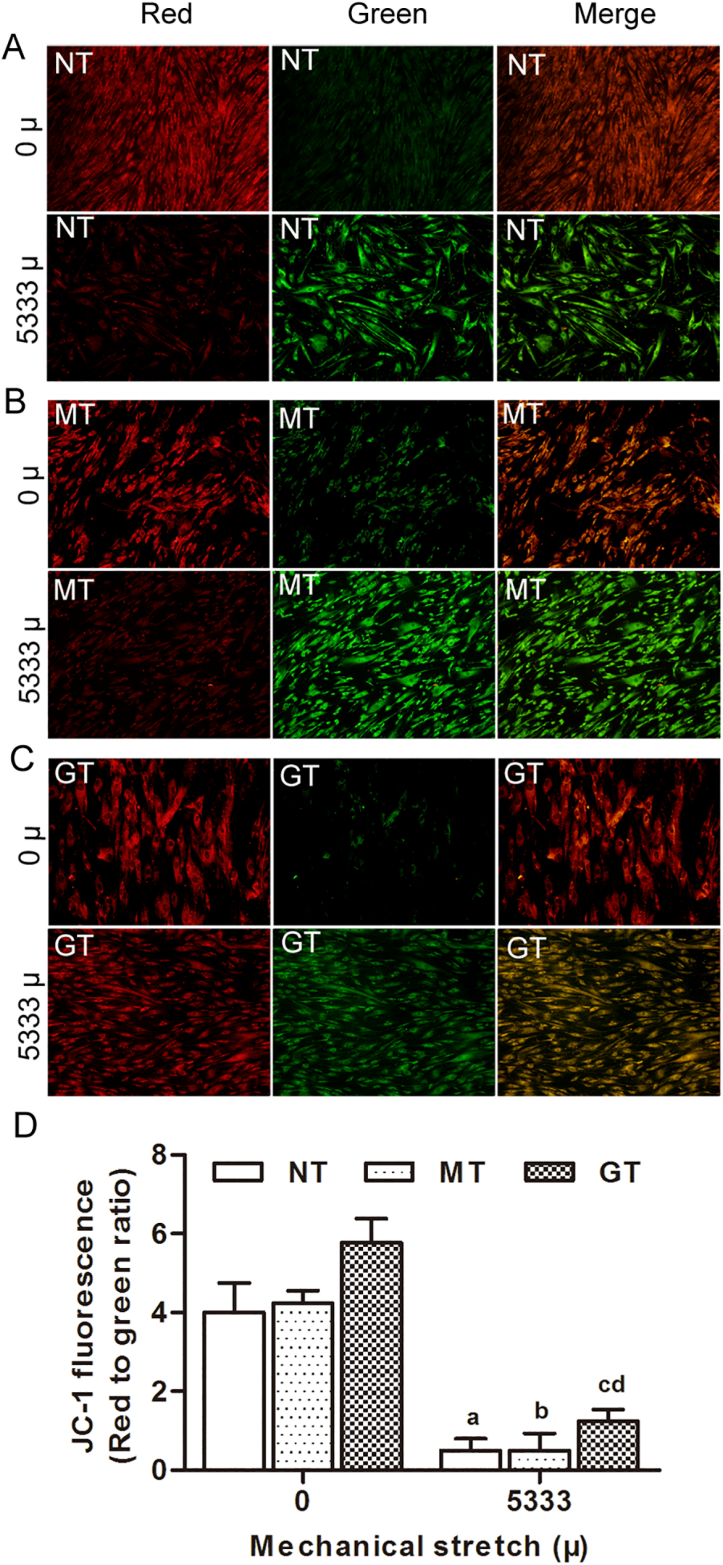
Assessment of the mitochondrial membrane potential by JC-1 staining. Representative fluorescence photomicrographs of in-situ JC-1-stained cells in various treatment groups (magnification, x200). (A-C) Three groups of hUSLFs (NT、MT、GT) were applied with mechanical strain of 0、5333μ, and then stained with JC-1 fluorescent probe. Images on the left show red fluorescence, indicating a high mitochondrial membrane potential; the middle panel shows green fluorescence, indicative of a loss of mitochondrial membrane potential. The right-hand panel shows merged red and green fluorescence. (D) Red and green fluorescence intensity was quantified by Image J software. The ratio of red to green fluorescence intensity is indicative of the mitochondrial membrane potential. Values are expressed as the mean fluorescence intensity ± standard deviation of five fields of view per group. One-way analysis of variance was performed, followed by an unpaired t-test. n = 3, a, *P*<0.05, vs. 0 μ, NT; b, *P*<0.05, vs. 0 μ, MT; c, *P*<0.05, vs. 5333 μ, NT; d, *P*<0.05, vs. 5333 μ, MT.

To investigate the role of GPX1 in mechanical strain induced apoptosis, GPX1-overexpressing group, mock-vehicle group and non-transfection group (**two control groups**), were applied mechanical strain of 0, 5333 μ at 0.1Hz for 4 hours. Apoptosis was assessed by Annexin V/PI double-staining and flow cytometry analysis (Fig 3). The quantified results showed that cell apoptosis rates increased in mechanical strain groups when comparing with unstretched groups. While in the three mechanical strain groups, cell apoptosis in the GPX1-overexpressing group was remarkably lower than that in the mock-vehicle group and non-transfection group (*p<*0.05). Mitochondrial membrane potential (ΔΨm) is another indicator for cell apoptosis. In normal cells, the JC-1 dye emitted a red fluorescence, indicating an intact ΔΨm. After mechanical stress loading, the red fluorescence of the JC-1 dye was weakened, and green fluorescence was enhanced among these three groups, quantitative analysis showed that the ratio of red to green fluorescence of cells decreased with loading strain when compared with the unstretched groups (*p<*0.05). The decreased red to green fluorescence ratio of JC-1 confirmed the decline in the ΔΨm. The GPX1-overexpressing group showed consistently higher ratio of red to green fluorescence than the two control groups When applying the 5333 μ mechanical strain (*p<*0.05) (Fig 4). These results indicated that GPX1 could protect hUSLFs from mechanical strain induced apoptosis.

### 3. Overexpression of GPX1 alleviated unbalanced-oxidation stress state in hUSLFs caused by mechanical strain

**Fig 5:**
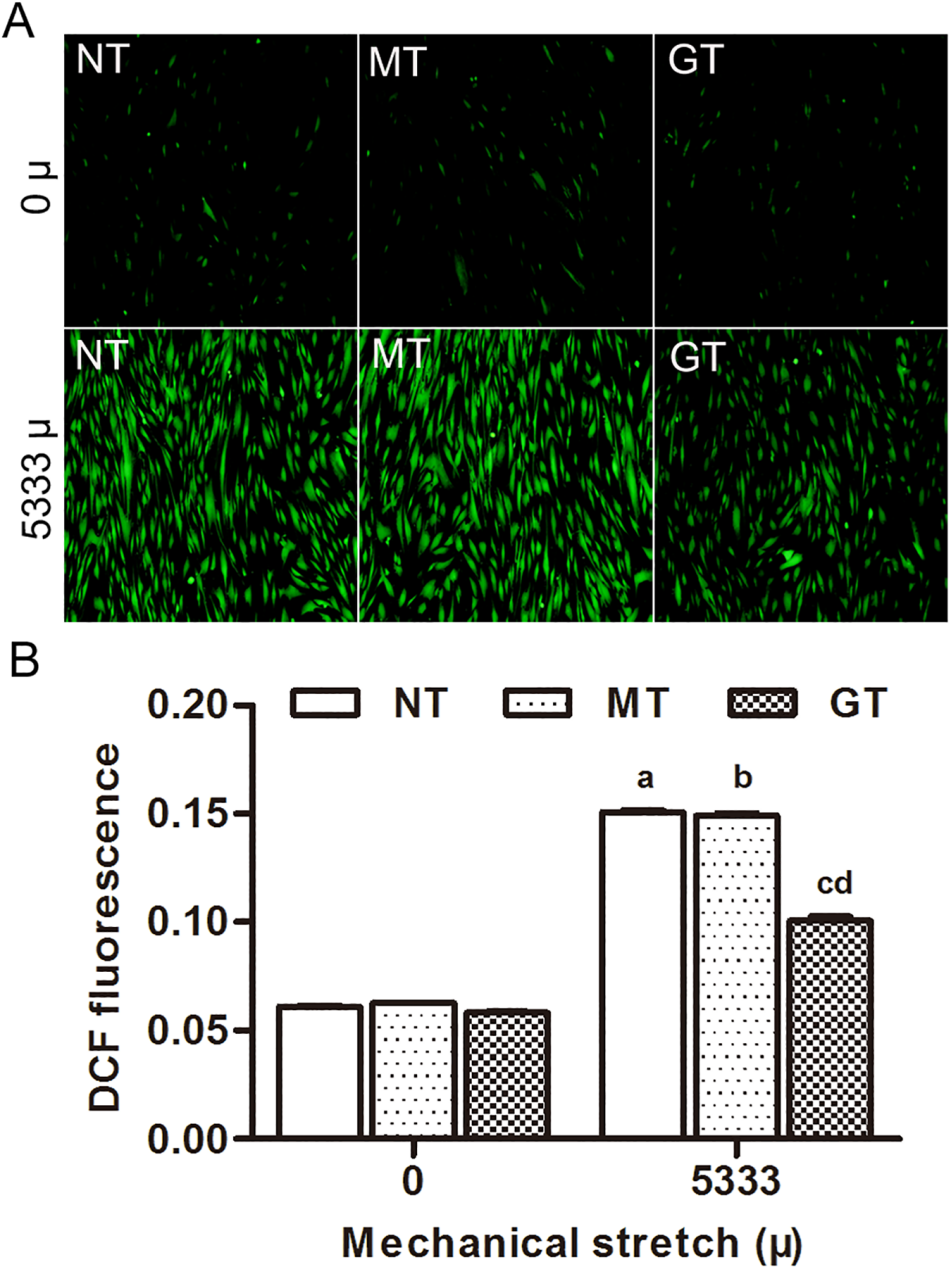
Microscopic images of ROS generation induced by mechanical stain using DCF-DA staining. (A) Three groups of hUSLFs (NT、MT、GT) were applied with mechanical strain of 0、5333μ, and then incubated with DCF-DA. The cells were observed under a fluorescent microscope (magnification, x200). (B) The intensity of DCF-mediated fluorescence was quantified using Image J software and reflected the levels of intracellular ROS. Values are expressed as the mean fluorescence intensity ± standard deviation of five fields of view per group. One-way analysis of variance was performed, followed by an unpaired t-test. n = 3, a, *P*<0.05, vs. 0 μ, NT; b, *P*<0.05, vs. 0 μ, MT; c, *P*<0.05, vs. 5333 μ, NT; d, *P*<0.05, vs. 5333 μ, MT. ROS, reactive oxygen species; DCF-DA, 2',7'-dichlorodihydrofluorescein diacetate.

To compare the level of intracellular ROS in the three groups of hUSLFs, a DCF-DA assay was performed following treatment with stress loading of 0 μ, 5333 μ at 0.1Hz for 4 h. As shown in Fig 5, the fluorescence intensity of the oxidized DCF, which indicates the induction of intracellular ROS increased in all three groups after 5333 μ mechanical strain loading compared to 0 μ mechanical strain. However, ROS level in GPX1-overexpressing group was significantly lower than that in the two control groups under the mechanical stress of 5333 μ (P<0.05).

**Fig 6:**
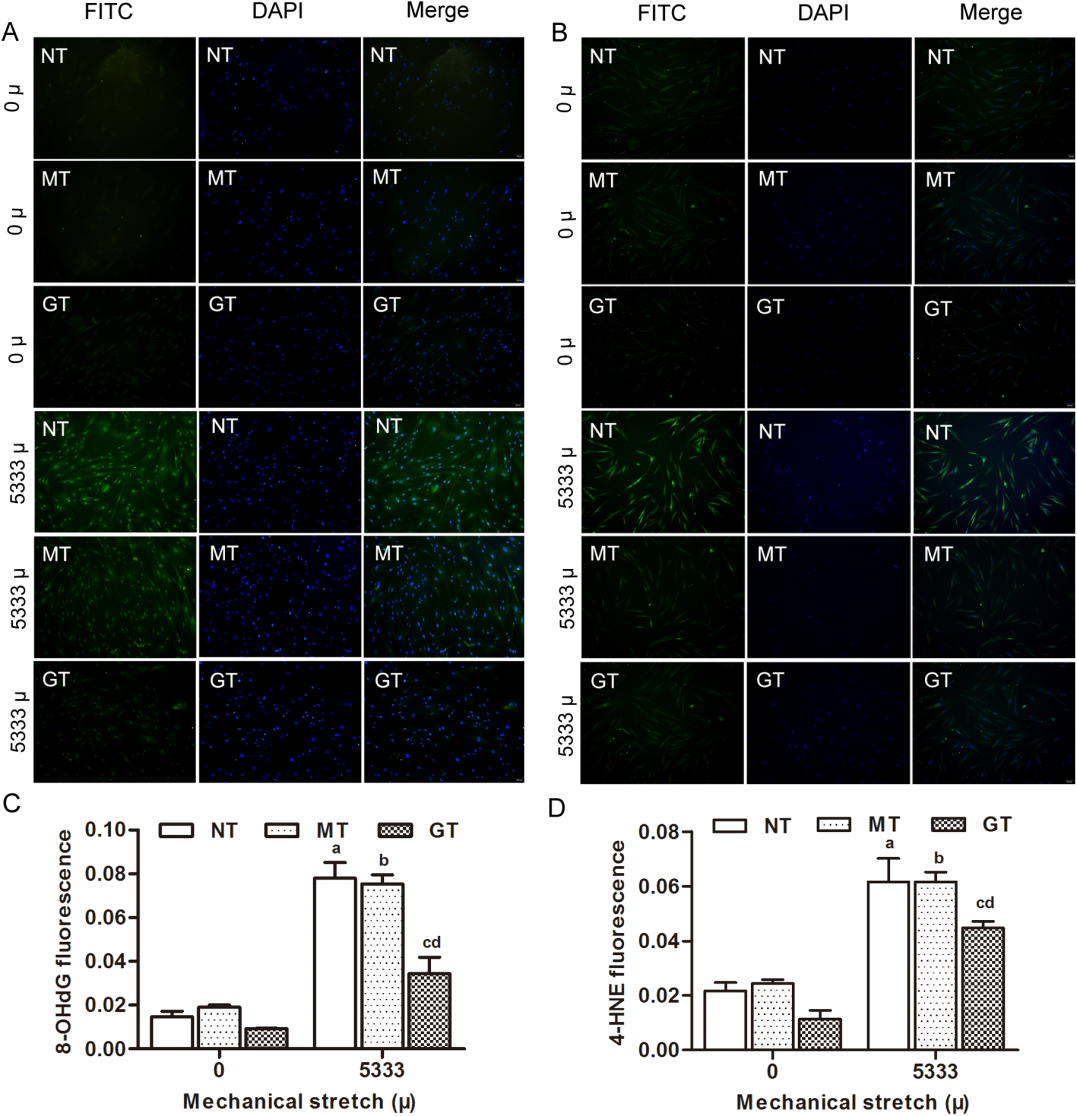
Microscopic images of 8-OHdG and 4-HNE production induced by mechanical strain using an immunofluorescent assay. (A) (8-OHdG) (B) (4-HNE) Three groups of hUSLFs (NT、MT、GT) were applied with mechanical strain of 0、5333μ, followed by incubation with 8-OHdG and 4-HNE antibodies, and then staining with DAPI. The cells were observed under a fluorescent microscope (magnification, x200). (C) (8-OHdG) (D) (4-HNE) Quantitative analysis based on fluorescence intensity, obtained using image-pro plus 6.0 software. Values are expressed as the mean fluorescence intensity ± standard deviation of five fields of view per group. One-way analysis of variance was performed, followed by an unpaired t-test. n = 3, a, *P*<0.05, vs. 0 μ, NT; b, *P*<0.05, vs. 0 μ, MT; c, *P*<0.05, vs. 5333 μ, NT; d, *P*<0.05, vs. 5333 μ, MT. 8-OHdG, 8-hydroxyguanosine; 4-HNE, 4-hydroxynonenal.

To clarify the oxidative damage due to the accumulation of intracellular ROS, the production of 8-OHdG and 4-HNE, two important biomarkers of oxidative damage, were examined by immunofluorescent assay. As shown in Fig 6, compared with the unstretched groups, the fluorescence intensity of 8-OHdG and 4-HNE increased when applying 5333 μ strain, and the intergroup differences were statistically significant (P<0.05). Interestingly, the fluorescence intensity of 8-OHdG and 4-HNE also decreased significantly in GPX1-overexpressing hUSLFs when subjected to 5333 μ strain compared to mock-vehicle group and non-transfection control group (P<0.05) (Fig 6). These results revealed that mechanical strain significantly elevated the levels of intracellular ROS and enhanced the oxidative damage production of 8-OHdG and 4-HNE in the hUSLFs, but overexpression of GPX1 could prevent mechanical strain-caused intracellular ROS, 8-OHdG and 4-HNE accumulation.

### 4. GPX1 improved the reestablishment of ECM in hUSLFs loaded with mechanical strain

**Fig 7:**
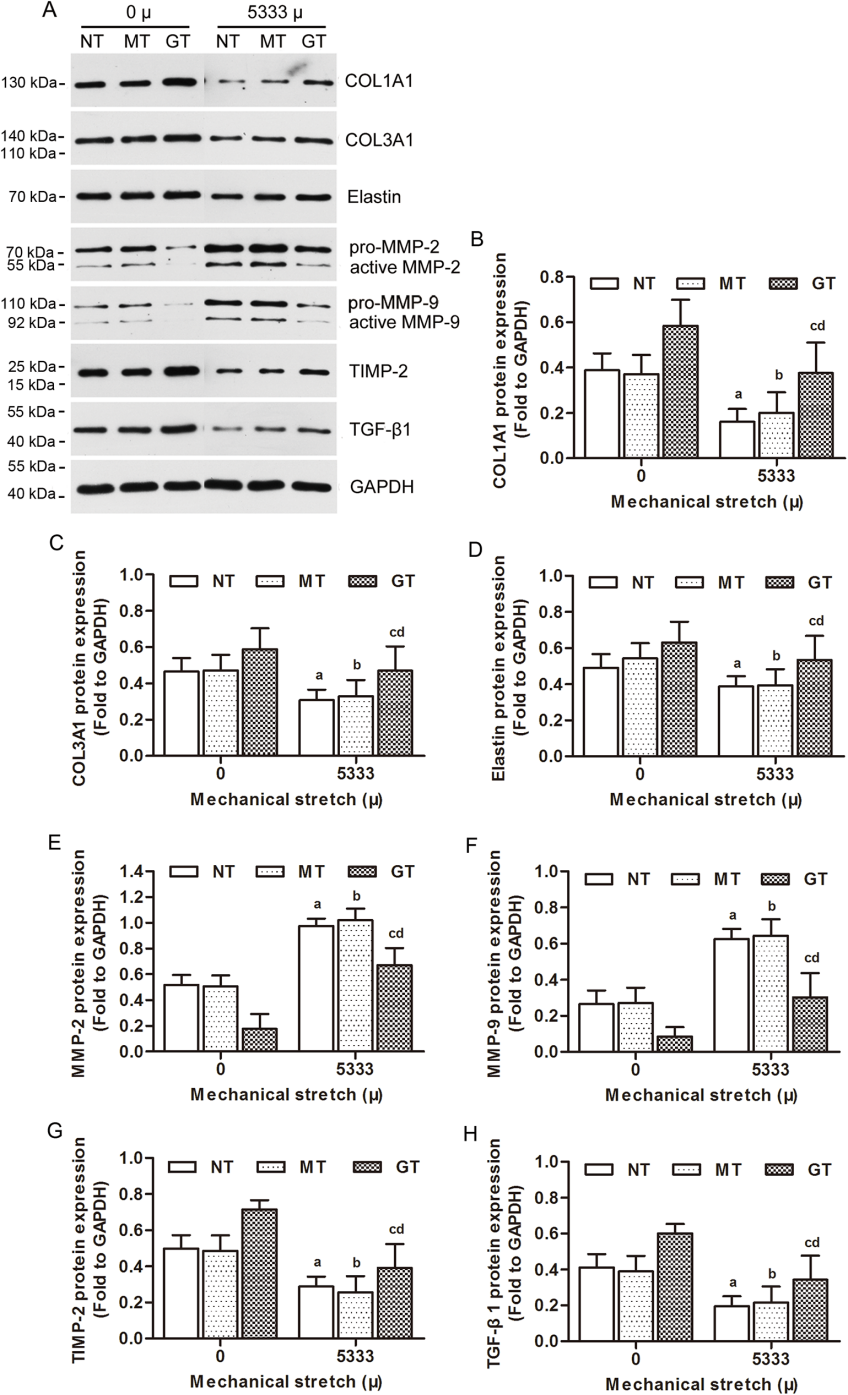
Effects of mechanical srain on ECM metabolism in human uterosacral ligament fibroblasts at the protein levels. (A) Three groups of hUSLFs (NT、MT、GT) were applied with mechanical strain of 0、5333μ, and then assayed by Western blot analyses. Quantitative analysis of (B) COL1A1, (C) COL3A1, (D) Elastin, (E) MMP-2, (F) MMP-9, (G) TIMP-2 and (H) TGF-β1 protein levels based on the bands of the Western blot. One-way analysis of variance was performed, followed by an unpaired t-test. n = 3, a, *P*<0.05, vs. 0 μ, NT; b, *P*<0.05, vs. 0 μ, MT; c, *P*<0.05, vs. 5333 μ, NT; d, *P*<0.05, vs. 5333 μ, MT. COL1A1, collagen, type 1, α1; COL3A1, collagen, type 3, α1; MMP-2, matrix metalloproteinase-2; MMP-9, matrix metalloproteinase-9; TIMP-2, tissue inhibitor of metalloproteinase-2; TGF-β1, transforming growth factor-β1.

**Fig 8:**
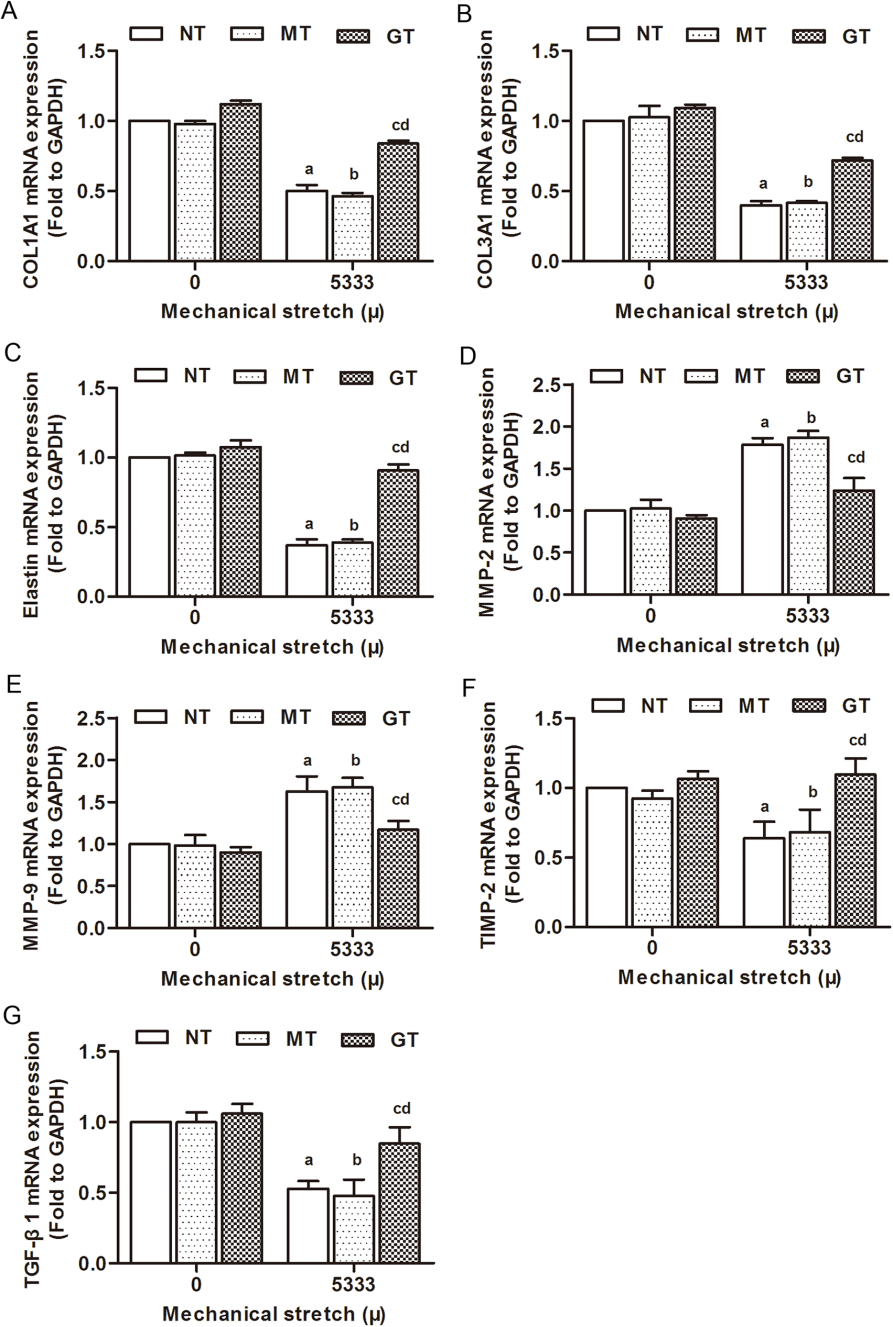
Effects of mechanical srain on ECM metabolism in human uterosacral ligament fibroblasts at the mRNA levels. Three groups of hUSLFs (NT、MT、GT) were applied with mechanical strain of 0、5333μ, and then the mRNA expression levels of (A) COL1A1, (B) COL3A1, (C) Elastin, (D) MMP-2, (E) MMP-9, (F) TIMP-2 and (G) TGF-β1 were examined by RT-qPCR analyses. One-way analysis of variance was performed, followed by an unpaired t-test. n = 3, a, *P*<0.05, vs. 0 μ, NT; b, *P*<0.05, vs. 0 μ, MT; c, *P*<0.05, vs. 5333 μ, NT; d, *P*<0.05, vs. 5333 μ, MT. COL1A1, collagen, type 1, α1; COL3A1, collagen, type 3, α1; MMP-2, matrix metalloproteinase-2; MMP-9, matrix metalloproteinase-9; TIMP-2, tissue inhibitor of metalloproteinase-2; TGF-β1, transforming growth factor-β1.

Type I、III collagen and elastin are main components of ECM in USL tissue, synthesized and secreted by fibroblasts. In order to investigate the effects of GPX1 on ECM metabolism in hUSLFs when subjected to mechanical stress, the collagen-related genes expression levels were assessed by quantitative PCR and Western blot. Our results showed that the mRNA and proteins levels of COL1A1, COL3A1 and elastin were significantly reduced in the 5333μ strain stretched fibroblasts when compared to 0μ strain groups (*p<*0.05 for each comparison). TGF-β1 is a multifunctional protein which promotes fibroblasts growth and ECM synthesis. The mRNA and protein levels of TGF-β1 were also significantly decreased in 5333μ stretched hUSLFs compared to unstretched cells (*p<*0.05, respectively). MMP-2 and MMP-9, which degrade collagen in hUSLFs, remarkably increased in mRNA and proteins (*p*<0.05, respectively). (Figs 7 and 8). There was a sharp decline in the mRNA and protein expression of TIMP-2 when the degree of mechanical stress increased from 0 to 5333μ, and the difference among these groups were significant (P<0.05) (Figs 7 and 8).

In the mechanical strain groups, mRNA and protein levels of COL1A1, COL3A1, elastin, TGF-β1 and TIMP-2 in GPX1-overexpression group cells significantly rose (*p*<0.05), and mRNA and protein levels of MMP-2 and MMP-9 decreased when compared to the control groups (*p*<0.05) (Figs 7 and 8).

Together, these results suggested that mechanical strain perturbs collagen metabolism in hUSLFs, and overexpression of GPX1 could prevent this process.

## Discussion

Many researches revealed that mechanical forces can activate OS signaling pathways. Cyclic mechanical forces were shown to significantly increase the levels of intracellular ROS in myoblasts in a dose-dependent manner[30]. If ROS can’t be effectively and safely eliminated, the excessive accumulation of intracellular ROS and high level of OS would cause abnormal metabolism, cell apoptosis and senescence [31]. GPX1 is generally distributed in many tissues and protects cells against oxidative damage[32, 33], and it is located in the cytoplasm and mitochondria in cells[32]. GPX1, which is a glutathione peroxidase, functions in the detoxification of hydrogen peroxide, specifically by catalyzing the reduction of hydrogen peroxide to H_2_O_2_ [32]. It is reported that GPX1 is the metabolic mediator of body Se in protecting against acute oxidative stress [34, 35]. It is possible that the lack of GPX1 exacerbates an endogenous age-dependent reduction in overall cellular function [35]. Together with our previous data that there was a decline in the expression of GPX1 in USL of women with POP [27], we try to figure out the exact role of GPX1 in the pathophysiology of POP.

In the present study, we applied mechanical strain to three groups of hUSLFs. Our data further confirmed that mechanical strain caused abnormalities in ECM metabolism via OS pathway in hUSFLFs, and it may play a significant role in the pathogenesis of POP, which was partially in accordance with previous data [18, 22, 23]. While in the presence of mechanical strain, compared with the non-transfection and mock-vehicle groups, the GPX1-overexpression group showed lower cell apoptosis, less mitochondria and oxidative damage, higher protein and mRNA levels of COL1A1, COL3A1, Elastin, TIMP-2 and TGF-β1, and less protein and mRNA levels of MMP-2, MMP-9. We found that GPx1-overexpression can not only protect hUSLFs from cell apoptosis, oxidative and collagen damage induced by mechanical stimulation, but also improve the remodeling of ECM in stretched fibroblasts. Our data revealed that GPx1 plays a significant role in regulating mechanical strain induced POP, which provides new theoretical basis for the antioxidants prevention and treatment of POP.

But our research has its potential limitations. First of all, we failed to confirm if GPx1 dysregulation in POP tissues was a consequence or a cause of the prolapsed and if OS is the only pathway involved in the mechanical force induced- the abnormal ECM metabolism. Secondly, there is no mature animal model for POP currently, so we also couldn’t jump to a conclusion that POP is caused by mechanical strain induced abnormalities in ECM metabolism via OS pathway without in vivo data. We could continue to figure out these questions and search whether there are other pathways involved in the pathogenesis of POP in our further study.
